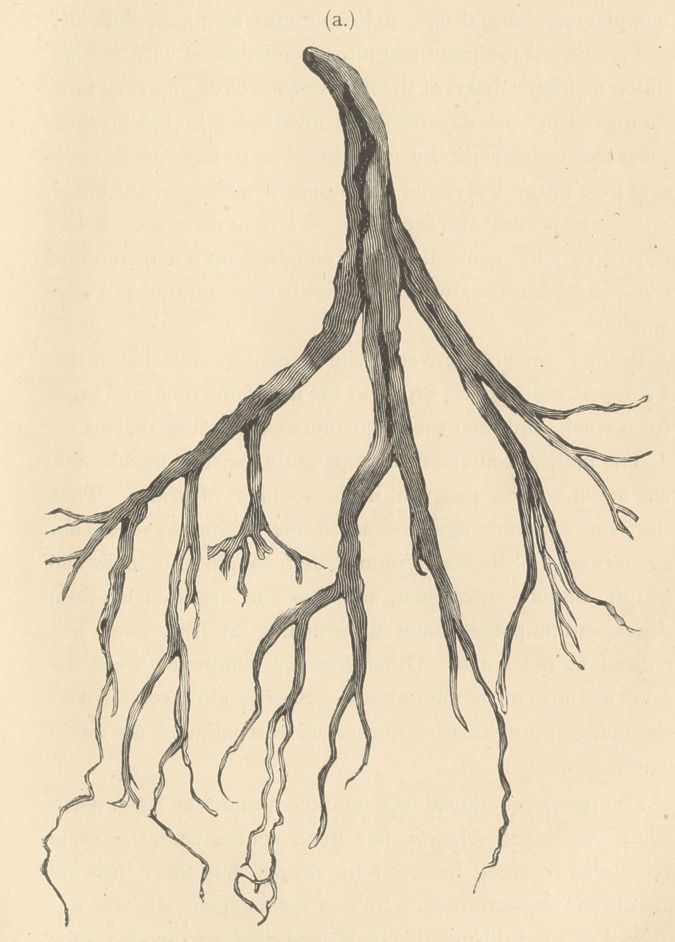# Pseudo-Membranous Bronchitis

**Published:** 1887-02

**Authors:** W. R. Patton

**Affiliations:** Charleston, Illinois


PSEUDO-MEMBRANOUS BRONCHITIS. *By* W. R. PATTON, M.D.

On the night of March 30, 1879, I was called to see Mrs. J—— in consultation with Dr. H., who had been attending the patient from some time in the prèceding November. From him, and from the friends of the patient, I learned that her trouble had begun with wheezing at night, very much as in croup, which at first occurred only occasionally. It increased in severity at each succeeding attack until she finally had to sit up in bed in order to breath. About January 1st, 1880, she began to have a troublesome cough. The cough was hoarse and wheezing, and she could lie down only between the paroxysms of coughing. These symptoms increased in severity until her life was despaired of. The fact that the patient was pregnant at this time increased the gravity of the case, but hope was entertained that if she could live until after confinement she would recover.

On March 30th, she was delivered of a healthy, well developed male child, but experienced no relief from her cough. My first visit was made on March 30th, 1880. I found the patient sitting up in bed, with no covering except her nightdress, and she could endure no more. She could not endure the doors or windows closed, although it was snowing, and a very cold, blustering night. She was the picture of death. She seemed almost bloodless; had no appetite, and could take only a swallow or two of anything at a time, without bringing on a paroxysm of coughing, which would continue as long as she had strength enough to cough. The pulse was 140 per minute and very weak. The urine was scanty, thick, and heavily loaded with solids, and there was a puffy œdematous condition of the eyelids and of the skin over the body. Examination of the lungs revealed no trouble except in the bronchial tubes. I could detect no lesion in the throat, and considered the trouble located only in the bronchial tubes; but in this opinion the attending physician did not concur; he regarded it as a pulmonary affection.

As a result of this difference in diagnosis the patient was placed under my professional care.

I prescribed a mixture of hive-syrup, syrup of ipecac, tincture of digitalis, and grindelia robusta, together with a tonic of carbonate of iron, gentian and Peruvian bark in gin, and pursued this course for three days. At the expiration of three days, to my astonishment, she coughed up a specimen of plastic material, (a.) which was a complete cast of the bronchial tubes from the largest to the smallest extremities.*

She was so much relieved by the expectoration of this cast that she could immediately lie down and sleep, for the first time in two months, for during that length of time, she had taken only short naps, and these in a sitting posture. On the next morning when shown the cast which had been expectorated, I was greatly astonished, and concluded that it was a different kind of bronchitis from what I had sup-

*The cast and a photograph of it were subsequently sent to Professor Ingals of Chicago. The accompanying sketch is from a photograph of it.

posed, and different from any I had ever seen. It has been my fortune not to see another, and only a repetition in the

(a.)

same case. She expectorated, during the progress of the case, twenty or thirty of these membranous casts, but none so complete as the first one, and none with so much difficulty.

I continued the treatment already mentioned with the addition of fifteen drops of the fluid extract of ergot every three hours during the day or when awake. I hoped by these measures to facilitate the expectoration, to improve the general tone of the body, and by the ergot acting on the blood-vessels, to prevent the formation of any more casts. Every day or two for some time she coughed up one or more of these casts, but they were more easily expectorated and were smaller.

In the course of six weeks she seemed so much better that I stopped visiting her, ordering the use of the tonic and ergot for a week, and then the discontinuance of all medicines.

The patient continued in apparently perfect health until she again became pregnant in the summer of 1881. Then the same condition again occurred, but neither so severe nor so alarming. She began immediately to take the tonic and cough mixture mentioned, and also the ergot in ten-drop doses, and no plastic material formed, or at least none was noticed in the sputa. Otherwise the symptoms were the same as those of the first attack. Shortly after confinement she made an excellent recovery, and discontinued the medicines.

The patient continued well until the summer of 1883, when about two months before her third confinement, the same symptoms returned followed by dropsy, but there was no membrane expectorated. In this confinement she was delivered of an eight-month's child, and died, two weeks after delivery, with general anasarca. A few days before her death she suffered greatly from dyspnœa, and was only able to breathe at all when sitting up, or standing with the assistance of friends. Membranes were evidently present, but she could not expectorate them. One singularity of this case was its recurrence during pregnancy. Prior to the last attack of pseudo-membranous bronchitis, the family had moved out of my neighborhood, and the patient was attended by another physician until almost a week before her death, when I was again called.

Charleston, Illinois.

## Figures and Tables

**Figure f1:**